# de.NBI Cloud federation through ELIXIR AAI

**DOI:** 10.12688/f1000research.19013.1

**Published:** 2019-06-10

**Authors:** Peter Belmann, Björn Fischer, Jan Krüger, Michal Procházka, Helena Rasche, Manuel Prinz, Maximilian Hanussek, Martin Lang, Felix Bartusch, Benjamin Gläßle, Jens Krüger, Alfred Pühler, Alexander Sczyrba

**Affiliations:** 1 Center for Biotechnology (CeBiTec), Bielefeld University, Bielefeld, North Rhine-Westphalia, 33104, Germany; 2 Institute of Computer Science, Masaryk University, Brno, 602 00, Czech Republic; 3 Department of Computer Science, University of Freiburg, Freiburg im Breisgau, Baden-Württemberg, 79110, Germany; 4 Omics IT and Data Management Core Facility (ODCF), German Cancer Research Center (DKFZ), Heidelberg, Baden-Württemberg, 69120, Germany; 5 Center for Bioinformatics (Applied Bioinformatics Group), University of Tübingen, Tübingen, Baden-Württemberg, 72076, Germany; 6 High Performance and Cloud Computing group (ZDV), University of Tübingen, Tübingen, Baden-Württemberg, 72074, Germany

**Keywords:** de.NBI, de.NBI Cloud, Cloud Computing, OpenID Connect, Life Sciences, ELIXIR, Authentication, Authorization

## Abstract

The academic de.NBI Cloud offers compute resources for life science research in Germany.

At the beginning of 2017, de.NBI Cloud started to implement a federated cloud consisting of five compute centers, with the aim of acting as one resource to their users. A federated cloud introduces multiple challenges, such as a central access and project management point, a unified account across all cloud sites and an interchangeable project setup across the federation. In order to implement the federation concept, de.NBI Cloud integrated with the ELIXIR authentication and authorization infrastructure system (ELIXIR AAI) and in particular Perun, the identity and access management system of ELIXIR. The integration solves the mentioned challenges and represents a backbone, connecting five compute centers which are based on OpenStack and a web portal for accessing the federation.This article explains the steps taken and software components implemented for setting up a federated cloud based on the collaboration between de.NBI Cloud and ELIXIR AAI. Furthermore, the setup and components that are described are generic and can therefore be used for other upcoming or existing federated OpenStack clouds in Europe.

## Introduction

In life sciences today, the handling, analysis and storage of enormous amounts of data is a challenging issue. For example, new sequencing and imaging technologies result in the generation of large scale genomic and image data. Hence, an appropriate IT infrastructure is necessary to perform analyses with such large datasets and to ensure secure data access and storage. The fully academic de.NBI Cloud, which is operated by the German Network for Bioinformatics Infrastructure (de.NBI), offers a solution to enable integrative analyses for the entire life science community and the efficient use of data in research. To a large extent, de.NBI Cloud will close the gap of missing computational resources for researchers in Germany.

Through a cloud federation concept, the five de.NBI Cloud sites, located in Bielefeld, Freiburg, Giessen, Heidelberg and Tübingen, are integrated into a single cloud platform, where the central access point of the federation is represented by the web-based de.NBI Cloud portal. The portal is a crucial part of the federated cloud concept and offers an easy entry point for de.NBI Cloud users and an important management tool for cloud and project administrators.

In collaboration with the European life-sciences Infrastructure for biological Information (ELIXIR) initiative, the de.NBI Cloud portal manages the authorization of users and offers single sign-on to all cloud centers. Perun ^1^, the identity and access management system used in ELIXIR, acts as a backbone for de.NBI Cloud. The interaction between the three entities: portal, cloud site and Perun, as well as the final federation concept that represents de.NBI Cloud, is explained in the following sections. This article demonstrates one way how to build a federated Cloud and the described components and concepts can be reused for upcoming or existing federated clouds.

## Methods

### Challenges

**Figure 1.**
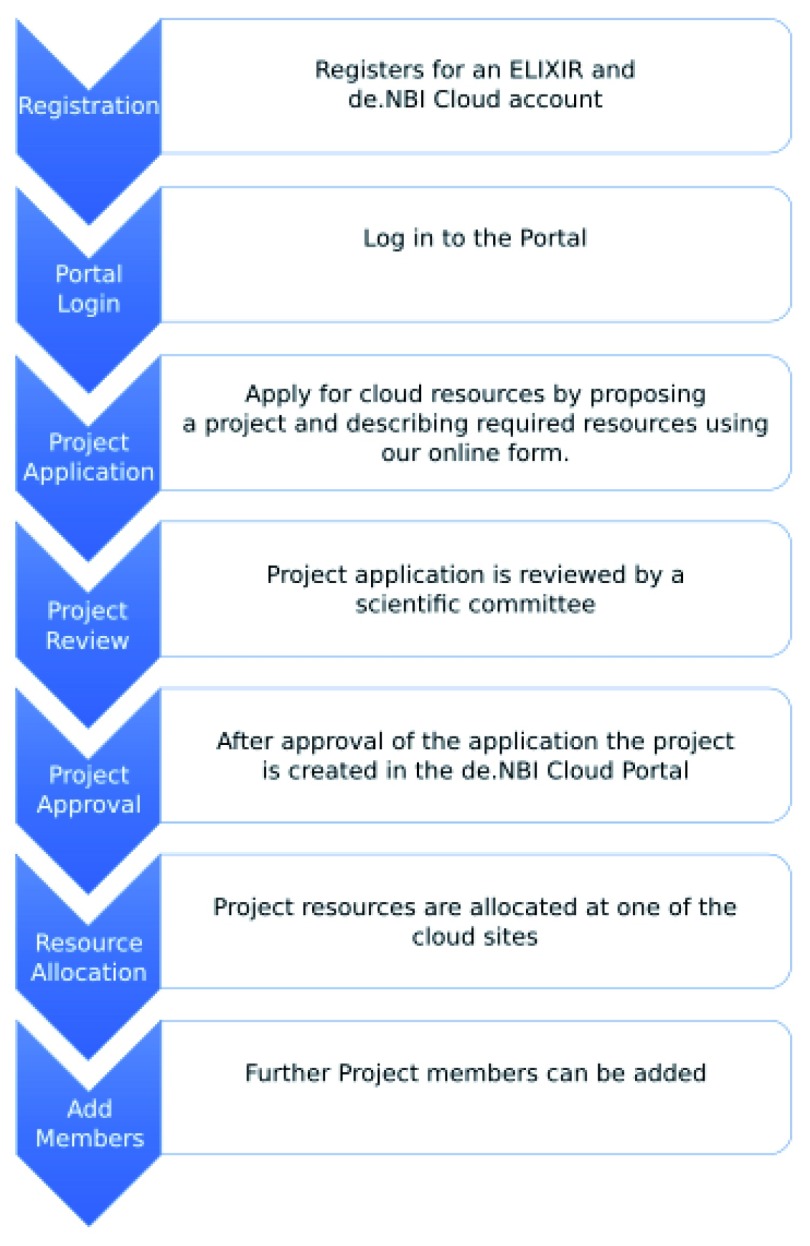
Project application process for de.NBI Cloud users.

***User workflow***. One of the main goals of de.NBI Cloud is to connect compute centers and the web-based portal through a federation concept. The workflow for a user who wants to start working in the cloud is depicted in Figure 1. The applicant must have an ELIXIR account and be a principal investigator at a German university or research institution. The application is submitted through a web form and is then reviewed by the cloud governance and an access committee. The cloud governance ensures the position of the applicant and determines, in consultation with the access committee, the appropriate resource allocation for a project. Once the project is approved, it can be hosted on one or a multiple of the five compute centers. The principal investigator can add any other user with an ELIXIR account during the project lifetime. Furthermore, they are responsible for any actions of their project members in the cloud.

This workflow implies multiple challenges, such as easy access to the portal and the centers, authorization of users with different roles and project coordination and configuration. The following sections explain these challenges and how they have been solved by the steps described in Implementation section.

***Easy access***. The de.NBI Cloud team wants to provide the easiest possible way to access the cloud. As described in the User Workflow section, the user must access the portal and also at least one of the compute centers, which at first glance means that multiple independent accounts must be created for every user. Creating user accounts and passwords means additional work for administrators and users.

***User authorization***. De.NBI users can have different roles. For instance, users can be members of a project and, if they are principal investigators (PI), they can be also managers of a project and add other users as members.

From the administration perspective, there is the compute center administrator, who maintains the OpenStack installation, and a scientific committee, who must approve PIs, approve projects and assign projects to different compute centers. In addition, de.NBI Cloud must ensure that the current user agreement according to the General Data Protection Regulation (GDPR) is accepted by all users.

***Project coordination and configuration***. A project can have a multitude of parameters that the user applies for in the de.NBI Cloud portal, which should be mirrored in the specific OpenStack project. Even though all cloud centers are OpenStack-based, they still differ in their version and to a certain extent in their setup, such as the usage of a jump host in one of the centers. In this scenario, the user must first ssh to a dedicated machine and then connect from it to a virtual machine provided by the cloud site.

Example project parameters are number of cores and amount of RAM, but also user specific parameters like an SSH public key, which allows the user to access a virtual machine. This also raises the question of how to transfer the configuration and the permissions of users to a specific OpenStack installation in an automatic way.

### Components

***OpenStack***. OpenStack is an open source Infrastructure as a Service (IaaS) system and allows users to define and start virtual machines. In the context of de.NBI, all cloud sites are operating an OpenStack installation. Keystone is the identity service of OpenStack and provides the authentication and authorization functionality of OpenStack.

***Perun***. Perun, as described in 1, is a system that provides functionality, covering management of the whole user life cycle in today’s e-Infrastructures, from user enrollment into the e-Infrastructure to user expiration. The Perun system supports management of virtual organizations, rights delegation, group management and enrollment management to provide flexible and easy to use user management. In comparison to ordinary identity management systems, Perun also provides service and access management.

***Single sign-on (SSO)***. SSO, according to 2, is the ability of a user to authenticate once to a single authentication authority and then access other protected resources without reauthenticating. This concept can be applied to de.NBI Cloud as follows: the authentication authorities are a university, Google, LinkedIn and ORCID. The protected resource is the de.NBI Cloud portal and the cloud sites.

***Shibboleth***. Shibboleth is an identity management system based on SAML ^3^, enabling single sign-on for users. In the context of de.NBI, universities are the identity providers, many of which use Shibboleth. Services like OpenStack installation use Shibboleth to allow users to log in with their home institutional account.

***ELIXIR Authentication and Authorization Infrastructure (AAI)***. ELIXIR AAI ^4^ allows researchers to log into services using identity providers available within eduGAIN - the interfederation of identity federation across the globe. Furthermore, ELIXIR AAI offers participating services additional functionality, such as group and attribute management, dataset authorization system or a multi-factor authentication. De.NBI Cloud is fully integrated with ELIXIR AAI. Ideally, the user is able to use his/her university account to access the cloud and any other service provided by de.NBI Cloud. If the university is not part of eduGAIN, alternatives such as Google, LinkedIn or ORCID can be used.

***OpenID Connect (OIDC)***. OpenID Connect, as explained on its website, is one of the SSO protocols and is placed on top of the OAuth 2.0 protocol ^5^. It allows clients like the de.NBI Cloud portal to receive information about end user and thereby to verify their identity based on the authentication performed by an Authentication Server.

### Implementation

The federation type of de.NBI Cloud comes closest to the definition of an Inter-Cloud Federation Framework (ICFF), as described in 6. In the case of de.NBI, the Inter-Cloud federation broker is represented by the de.NBI Cloud portal, which coordinates and allocates resources.

In de.NBI Inter-Cloud Federation Framework, the OIDC authorization framework is used for transferring data between the following entities: 1) user agent and the de.NBI Cloud portal, 2) the portal and Perun, 3) user agent and OpenStack.

**Figure 2.**
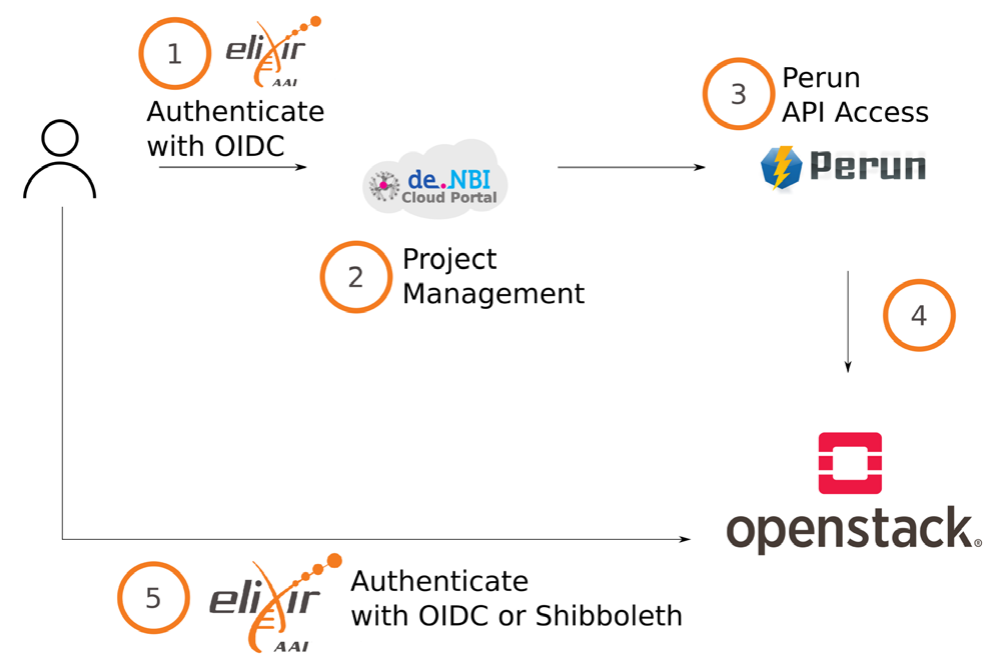
The user workflow which is implemented by the de.NBI Cloud Federation. 1. User logs in to the portal and applies for a project. 2. The Virtual Organisation manager configures the project. 3. Project configuration data is saved in Perun. 4. The project data is propagated to an OpenStack installation.

The implementation of each step is depicted in Figure 2. Once the user logs into the portal ( **Step 1**), they must be authenticated against an identity provider enabled for ELIXIR AAI and also registered for the de.NBI Cloud virtual organization. By logging into the portal, data, such as information about the identity provider (e.g: university or Google account) and the position of the user in the institution, can be retrieved by the portal. Whereat the position (e.g. employee, member, student) can just be accessed if an identity provider of an university was used.

Using the token provided by OIDC, the portal can make API calls to Perun and save data, such as de.NBI Cloud-specific project configuration ( **Step 2**). Furthermore, de.NBI Cloud reuses Perun roles and translates them into de.NBI Cloud specific user roles (see Table 1).

**Table 1.** The translation of Perun-specific roles to de.NBI Cloud-specific roles.

Perun	de.NBI Cloud
Group Manager	Project Manager
Group Member	Project Member
Facility Manager	Administrator
Virtual Organisation Manager	Access Committee

All requests are made on behalf of the user, with their own access token ( **Step 3**). The user role in Perun influences the data shown and functionality offered on the portal. In addition, any information about a de.NBI Cloud project can be saved in group-specific attributes in Perun. A number of defined attributes are listed in Table 2. For example, a project manager in de.NBI Cloud can add ELIXIR users to their project because he or she is also group manager in Perun. Each project is assigned programmatically by the portal to a facility whereby a facility in Perun is a compute center in de.NBI Cloud.

**Table 2.** An incomplete list of attributes provided by Perun. Attributes marked with (*) are not yet used by the portal or the PerunKeystoneAdapter.

Attribute Name	Attribute Description	Data Type
Project Perun ID	Autogenerated project ID	String
Project Name	Name of the project.	String
Project Description	Description of the project.	String
Project Members	All members of this project (e.g. ELIXIR ID, ELIXIR login name, ssh public key)	Map
Newsletter	Newsletter about recent cloud activities	Boolean
Cores Limit	Number of cores allowed for this project	Number
RAM Limit	Amount of RAM allowed for this project	Number
Number of Floating IPs	Number of Floating IPs allowed for this project	Number
*Number of granted credits	Number of cloud credits granted for this project	Number
*Number of used credits	Number of cloud credits used in this project	Number
Number of VMs	Number of VMs allowed for a project	Number
Volume Limit	Maximum size of all volumes combined for a project	Number
Volume Counter	Number of volumes allowed	Number
Object Storage *	Allowed maximum size of all objects combined of one project saved in the object storage	Number

A facility can propagate its data over HTTPS or SSH ( **Step 4**) as soon as any project-related data changes occur, such as changes to project members or the project quota. The propagated data is in JSON format and consists of project specific configurations, quotas and user-specific SSH keys and ELIXIR IDs. This data does not contain OpenStack-specific information and could be translated into any Infrastructure as a Service System. In the case of de.NBI Cloud, we built an open source tool called PerunKeystoneAdapter, which translates the data into Keystone. It can be extended and customized to the setup of the specific cloud site, such as setting ELIXIR login names on a jump host. Once the ELIXIR ID of a user is saved in Keystone, they can enter OpenStack through Shibboleth or OIDC, depending on the implementation used in the OpenStack setup ( **Step 5**). Finally, the fact that every user must be registered for the de.NBI Cloud virtual organization is a useful administrative feature. If a user agreement is updated, it must be first accepted by a user before they can access any de.NBI Cloud related service (portal, cloud site).

## Conclusions

De.NBI Cloud represents a federated cloud, allowing users to access the web portal and all five cloud centers with just one account. With the described concept, it is easy to guide the user from the application process until the start of a virtual machine. From the de.NBI Cloud developers perspective, the implementation is made of loosely coupled components (portal, Perun, cloud center) that makes it easy to extend the cloud with additional centers or customize certain components of any cloud site setup. Even the usage of a different Infrastructure as a Service system could be easily implemented without modifying the portal, Perun or any of the existing cloud sites, which allows for the adaption of our concept by other federated clouds. Additionally, administrative features, such as the necessary approval of project applicant positions or ensuring the agreement of users of all cloud sites to the current policy, can be done with minimal effort.

In summary, ELIXIR AAI represents the backbone of the de.NBI Cloud federation and, in conjunction with OpenID Connect, it enables features that are promising for the future. Two of those features are explained in the Outlook section.

### Outlook

The concept of OpenID Connect in the context of ELIXIR AAI allows an integration of "third party" software and to delegate permissions to them to access certain cloud sites. In the context of de.NBI, it means that de.NBI services can be registered for de.NBI Cloud and, by using OpenID Connect, the service can outsource workloads on demand to the Cloud.

Furthermore, ELIXIR AAI enables the design of a permission API to restrict access to sensitive data, such as human data. A proof of concept is already implemented by the CSC cloud, in which they demonstrate how a virtual machine can access datasets of the European Genome-phenome Archive (EGA).

## Data availability

No data are associated with this article
