# Supplementary material for: de.NBI Cloud federation through ELIXIR AAI
Source: F1000Res. 2019 Jun 10;8:842. [Version 1] doi: 10.12688/f1000research.19013.1 (PMC6635982; doi:10.12688/f1000research.19013.1)
Supplement: Peer review history

# Peer review: Reviewer response for version 1

**DOI:** 10.5256/f1000research.20838.r49754

**Published:** 2019-07-15

**Authors:** Volker P. Brendel

**Affiliations:** 1 Department of Biology, Indiana University Bloomington, Bloomington, IN, USA

The article describes the implementation of de.NBI Cloud, which gives integrated access to currently five compute centers through one portal. The end user needs are well stated, and the technical challenges to meet those needs are clearly described. It is outside of my expertise to comment on the technical soundness of the implementation, and no user survey statistics are provided to assess user satisfaction.

 As de.NBI Cloud is clearly not the first implementation of such a system, I am surprised that the authors don't discuss precedents. For example, I have been a happy user of XSEDE, which is probably the largest similar network. Within XSEDE, Jetstream provides access to virtual machines (run off machines at either TACC or Indiana University). I believe the backend implementation is rather similar, but maybe that should be discussed in the article.

I confirm that I have read this submission and believe that I have an appropriate level of expertise to confirm that it is of an acceptable scientific standard.

# Peer review: Reviewer response for version 1

**DOI:** 10.5256/f1000research.20838.r49751

**Published:** 2019-07-02

**Authors:** Dixie Baker

**Affiliations:** 1 Martin, Blanck & Associates, Alexandria, VA, USA

The abstract claims that this paper “explains the steps taken and software components implemented for setting up a federated cloud based on the collaboration between de.NBI Cloud and ELIXIR [Authentication and Authorization Infrastructure (AAI)].” Unfortunately, the paper falls short of its promise. Rather, the paper seems to be an unnecessarily complex and confusing description of both the problem being addressed and the approach taken for the de.NBI.

 Fundamental objectives that de.NBI, and others seeking to federate identity and authorization across multiple service providers, need to address are 1) to gain assurance that an individual user is the identity he/she asserts (i.e., user authentication); 2) to obtain reliable information about the security attributes of each authenticated individual (i.e., authorization); and 3) to share an authenticated identity and authorization attributes with other trusted nodes in a network (i.e., identity federation) to the level of assurance required by each node.    

 Although methods and standards used for authenticating identity and sharing security attributes (e.g., SAML, OIDC) differ in ways described in their respective specifications, every method relies on trust established through metadata (e.g., client_id, expiration, scope), and digitally signed (and optionally encrypted) tokens (e.g., JWT, SAML2). The key challenge that de-NBI (and other organizations) is facing is how to achieve syntactic and semantic interoperability among these methods so that data service providers can confidently make access-control decisions based on the tokens they receive from their respective trusted service providers. This paper never succinctly states these objectives and central challenge.  

 The “software components” listed include some basic concepts (e.g., “Single sign-on”), some components that are part of other components (e.g., Perun, Elixir AAI), and some components that include services provided by other components (e.g., OpenStack’s Keystone). How these components operate together for de.NBI is not explained. The workflow given in Figure 2 is unclear and confusing – two authentication paths (labelled paths 1 and 5) are shown, both involving Elixir and both including OIDC. It appears that project configuration data are saved only through path 1. The explanation of OIDC “as explained on its website” is superficial at best. Both OpenID Connect (OIDC) and OAuth 2.0 are worthy of more complete explanation.  

 Many identity brokers capable of providing single sign-on service across SAML and OIDC exist – including the broker used by Elixir itself. I fail to see the value that this paper would provide to its readers. 

I confirm that I have read this submission and believe that I have an appropriate level of expertise to state that I do not consider it to be of an acceptable scientific standard, for reasons outlined above.

# Peer review: Reviewer response for version 1

**DOI:** 10.5256/f1000research.20838.r49755

**Published:** 2019-07-02

**Authors:** Enol Fernández-del-Castillo

**Affiliations:** 1 EGI Foundation, Amsterdam, The Netherlands

This article presents the de.NBI IaaS federation which brings together 5 different OpenStack installations for researchers in life science. The system provides single-sign on by leveraging federated identity technology such as SAML and OpenID Connect with ELIXIR AAI, an established source of identity in the life science community in Europe. 

- Access to the federation is managed by a central de.NBI portal that manages users in projects using Perun and synchronises that information to the different OpenStack providers using an Open Source tool. Source code of the portal is not available (or not references in the article at least), but it may be interesting for other similar initiatives to replicate the setup. Authors should consider adding a reference to the source code if available.
- Description of the federation is clear. Comparison with other existing cloud federation initiatives would improve the article. 
- de.NBI is sometimes written as De.NBI, this should be consistently capitalised.
- Consider using the "Software Tool Article" instead of "Method Article". 

I confirm that I have read this submission and believe that I have an appropriate level of expertise to confirm that it is of an acceptable scientific standard.
